# Associations Between Cardiovascular Health (Life's Essential 8) and Mental Disorders

**DOI:** 10.1002/clc.70019

**Published:** 2024-09-24

**Authors:** Yudi Xu, Wenjing Ning, Yuyuan Zhang, Yuhao Ba, Huimin Liu, Long Liu, Libo Wang, Chunguang Guo, Hui Xu, Siyuan Weng, Zhaokai Zhou, Zongao Cai, Hongxuan Ma, Ge Zhang, Yanjie Jia, Xinwei Han

**Affiliations:** ^1^ Department of Neurology The First Affiliated Hospital of Zhengzhou University Zhengzhou Henan China; ^2^ School of Nursing The Hong Kong Polytechnic University Hung Hom Hong Kong; ^3^ Department of Interventional Radiology The First Affiliated Hospital of Zhengzhou University Zhengzhou China; ^4^ Department of Hepatobiliary and Pancreatic Surgery The First Affiliated Hospital of Zhengzhou University Zhengzhou Henan China; ^5^ Department of Endovascular Surgery The First Affiliated Hospital of Zhengzhou University Zhengzhou Henan China; ^6^ Department of Urology Surgery The First Affiliated Hospital of Zhengzhou University Zhengzhou Henan China; ^7^ Department of Vascular Surgery The First Affiliated Hospital of Zhengzhou University Zhengzhou Henan China; ^8^ Department of Kidney Transportation The First Affiliated Hospital of Zhengzhou University Zhengzhou Henan China; ^9^ Department of Cardiology The First Affiliated Hospital of Zhengzhou University Zhengzhou Henan China; ^10^ Interventional Institute of Zhengzhou University Zhengzhou Henan China; ^11^ Interventional Treatment and Clinical Research Center of Henan Province Zhengzhou Henan China

**Keywords:** anxiety, cardiovascular health, depression, Life's Essential 8, NHANES

## Abstract

**Background:**

Mental health was closely associated with cardiovascular disease (CVD). We aimed to investigate the association between cardiovascular health (CVH), as defined by Life's Essential 8 (LE8), and the presence of depression and anxiety.

**Hypothesis:**

We hypothesized that CVH, as defined by LE8, was negatively associated with the prevalence of depression and anxiety.

**Methods:**

A cross‐sectional study was conducted on participants (≥ 20 years old) from the National Health and Nutrition Examination Survey (NHANES). The LE8 score (ranging from 0 to 100) was composed of the health behavior score and the health factor score, which were further categorized into three levels as follows: low (0–49), moderate (50–79), and high (80–100). Weighted multivariable logistic regressions and restricted cubic splines were utilized to assess the association between LE8 and mental disorders.

**Results:**

Among the 13 028 participants included in this research, 1206 were determined to have depression symptoms and 2947 were determined to have anxiety symptoms. In the weighted and adjusted model, LE8 was negatively associated with the prevalence of depression (odds ratio [OR], 95% confidence interval [CI]: 0.61, 0.58–0.65) and anxiety (OR, 95% CI: 0.78, 0.75–0.81). Furthermore, a nonlinear dose–response relationship was observed between LE8 and anxiety.

**Conclusions:**

CVH defined by the LE8 was independently and negatively associated with the prevalence of depression and anxiety. Interventions targeting LE8 components may improve both CVH and mental health.

## Graphical abstract

Cardiovascular health defined by the LE8 was independently and negatively associated with the prevalence of depression and anxiety. Interventions targeting LE8 components may improve both cardiovascular health and mental health.

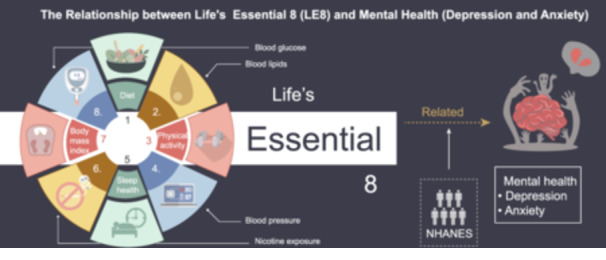

**Abbreviations**
AHA: American Heart Association
BMI: body mass index
CI: confidence interval
CVD: cardiovascular disease
CVH: cardiovascular health
HEI‐2015: Healthy Eating Index 2015
HPA: hypothalamic‐pituitary‐adrenal axis
LE8: Life's Essential 8
LS7: Life's Simple 7
NHANES: National Health and Nutrition Examination Survey
OR: odds ratio
PHQ‐9: Patient Health Questionnaire‐9
RCS: restricted cubic spline

## 1 Introduction

As the most common psychiatric disorder worldwide, depression and anxiety are the leading causes of disability and premature mortality [1, 2, 3]. Different from normal mood fluctuations, depression is characterized by persistent feelings of sadness and lack of motivation, while anxiety is characterized by persistent, overwhelming, and acute worries [1, 4]. These mental disorders could severely impede patients' daily lives and impose enormous pressure on the social economy [5]. Thus, proactive prevention of depression and anxiety has emerged as a public health priority as well as a major challenge.

Notably, depression and anxiety are not merely mental disorders but also interplay with physical health, especially cardiovascular health [6]. Dhingra et al. demonstrated that the incidence of developing moderate‐to‐severe depression was 2.97 times higher in participants with four cardiovascular disease (CVD) risk factors than in those with no CVD risk [7]. Additionally, a prospective cohort study illustrated the close link between anxiety and myocardial infarction [8]. Therefore, further exploring the relationship between cardiovascular health and depression and anxiety may be beneficial in terms of preventing these mental disorders in clinical settings.

To assess cardiovascular health, the American Heart Association (AHA) published a simple 7‐item tool in 2010, termed Life's Simple 7 (LS7), which encompassed seven crucial metrics as follows: three behavioral metrics (smoking, physical activity, and diet) and four factor metrics (body mass index [BMI], blood pressure, blood glucose, and total cholesterol) [9]. Extensive evidence has established that individuals with more ideal LS7 metrics presented better cardiovascular health and a lower risk of CVD [10, 11]. Previous studies also examined the correlation between LS7 and depression, discovering that an optimal level of LS7 was associated with a decreased incidence of depression [12]. Nevertheless, LS7 gradually demonstrated its limitations on the scoring system and psychological health has been acknowledged as foundational to cardiovascular health [13]. Accordingly, AHA issued an upgraded version of LS7 termed Life's Essential 8 (LE8), which featured a new scoring algorithm and incorporated sleep health as the eighth cardiovascular health metric [13]. The new scoring algorithm of LE8 improved the sensitivity for assessing the health of cardiovascular system, enabling better detection of interindividual variances and intraindividual fluctuations over time [14]. Thus, LE8 could more accurately mirror cardiovascular health when exploring its relationship with depression and anxiety. However, the association between the newly launched LE8 and depression and anxiety is yet unclear.

To fill the research gap, a nationally representative cohort from the National Health and Nutrition Examination Survey (NHANES) was leveraged to assess the association between cardiovascular health defined by LE8 and depression and anxiety. The findings of this study may potentially direct the prevention of depression and anxiety from a clinical perspective and shed light on the intricate links between mental and physical health.

## 2 Methods

### 2.1 Study Population

**Figure 1:**
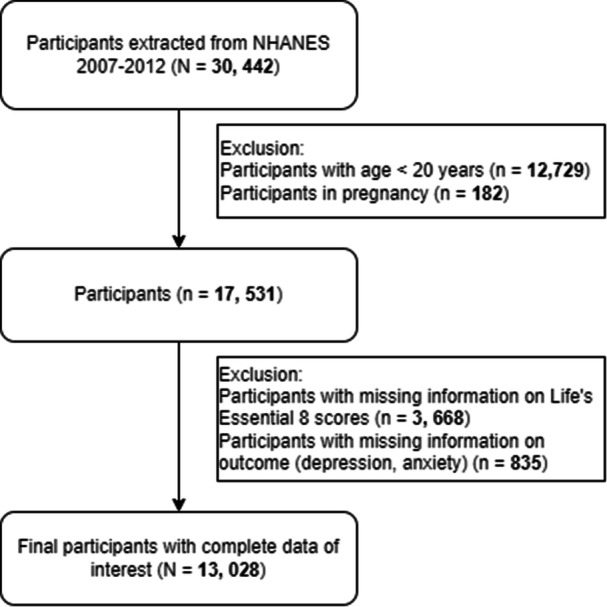
Flowchart of participants selection. NHANES, National Health and Nutrition Examination Survey.

NHANES is an ongoing cross‐sectional research program designed to evaluate people's health and nutrition status in the United States. The program was conducted in 2‐year cycles and employed a stratified multistage sampling design to obtain representative samples of US residents. The Centers for Disease Control and Prevention (CDC; https://www.cdc.gov/nchs/nhanes/index.htm) provided detailed procedures and data for NHANES, which mainly consisted of questionnaire and examination data collected from telephone or in‐home interviews and NHANES Mobile Examination Center (MEC) visits, respectively. All participants were required to sign informed consent at the point of enrollment. The current study utilized three waves of NHANES data spanning from 2007 to 2012, focusing on participants 20 years of age or older (*n* = 17 713). Participants who were pregnant were excluded (*n* = 182). Among the initial pool of participants, participants with incomplete information on LE8 metrics were excluded from the study (*n* = 3668). Additionally, those without outcome data were removed from the analysis (*n* = 835). Ultimately, 13 028 participants with complete data were included in the analysis (Figure 1).

### 2.2 Assessment of Cardiovascular Health Metrics

Cardiovascular health was assessed by the LE8 score, a sensitive indicator proposed by AHA. The components of the LE8 score comprised four health behavior metrics (diet, physical activity, nicotine exposure, and sleep health) and four health factor metrics (BMI, blood glucose, blood lipids, and blood pressure). Diet score was assessed using the Healthy Eating Index 2015 (HEI‐2015) [13, 15]. Information regarding physical activity (minutes per week), nicotine exposure, sleep health (average hours per night), medication history, and diabetes history was collected from a self‐reported questionnaire. BMI was calculated through height and weight. Fasting blood glucose and HbA1c were utilized to assess the blood glucose score. Non–high‐density lipoprotein (non–HDL) cholesterol was leveraged to determine blood lipid levels. Blood samples were collected from MEC and analyzed by central laboratories to obtain information on the blood biochemical indicators.

The scoring details for each metric were discussed in the study conducted by AHA [13]. Individual cardiovascular health metric was scored separately on a scale of 0–100 within the scoring criteria, and the LE8 total score was calculated by taking the unweighted mean of the eight cardiovascular health metrics. Higher LE8 scores represented better cardiovascular health. Furthermore, the LE8 total score was categorized into three levels by cut‐off points as follows: low group (0–49), moderate group (50–79), and high group (80–100) [13]. Analogically, the LE8 health behavior score and LE8 health factor score were calculated by dividing the sum of their respective components by four and were also categorized into three levels using the aforementioned cut‐off points.

### 2.3 Outcomes

Depression symptom was assessed by the Patient Health Questionnaire (PHQ‐9), which encompassed nine items based on the DSM‐IV diagnostic criteria and demonstrated high reliability and validity in previous studies [16]. The PHQ‐9 score could reflect depressive status in the last 2 weeks and was calculated by summing the scores of nine items (ranging from 0 “not at all” to 3 “nearly every day”), yielding a total score ranging from 0 to 27 [16]. In the present study, we leveraged PHQ‐9 score ≥ 10 as the cut‐off point for identifying depression, which has been demonstrated with both sensitivity and specificity of 88% [17, 18].

Anxiety status was assessed via the Computer‐Assisted Personal Interviewing (CAPI) system, in which participants were asked “During the past 30 days, for about how many days have you felt worried, tense, or anxious?” Participants reported that 7 or more anxious days were defined as anxiety status. This assessment was based on 14‐item Healthy Days Measures set by the CDC and was encompassed in health‐related quality‐of‐life (HRQoL) assessments, which featured a moderate to excellent reliability of surveillance questions [19].

### 2.4 Covariates

Other covariates encompassed age, sex, race (Mexican American, Other Hispanic, non‐Hispanic White, non‐Hispanic Black, other race/multiracial), marital status (coupled, single, or separated), poverty, and education (> high school, high school, and < high school). Age was further categorized into two groups (young adults and old adults) based on the mean age [12]. Poverty was defined as the ratio of monthly family income to poverty level [20] and categorized into the poverty group (≤ 2.13) and non‐poverty group (> 2.13) according to the mean.

### 2.5 Statistical Analysis

The NHANES employs a complex, multistage probability sampling design to ensure that the data collected are representative of the civilian, noninstitutionalized US population. NHANES provides sampling weights for each participant, which are derived based on the inverse probability of selection. These weights account for the oversampling of certain population subgroups (such as minority groups and the elderly), nonresponse adjustments, and post‐stratification adjustments to align the survey sample with the US Census demographic distributions. Given the complex multi‐probability sample weighting design of NHANES data, all analyses in the present study were conducted on nationally representative estimates generated by weighting the sample. Continuous variables were described by weighted mean and standard errors, whereas categorical variables were described by weighted participant numbers and percentages. Comparison of characteristics by depression status was performed via *t*‐test for continuous variables and chi‐squared test for categorized variables.

The survey‐weighted multivariable logistic regression was utilized to explore the association between cardiovascular health metrics and the risk of depression and anxiety. The model was adjusted for six factors as follows: age, sex, race, marital status, poverty, and education. We also calculate the *p* for a trend of categorical variables by taking the median of each category as a continuous variable in the multivariable logistic regression model [21]. Restricted cubic spline (RCS) was employed to investigate the potential nonlinear relationship between LE8 and the outcome variable in our regression analyses. Unlike traditional linear regression, which assumes a straight‐line relationship between the predictor and outcome, RCS allows for greater flexibility by fitting a smooth, piecewise polynomial function to the data. To investigate the effect of demographically relevant divergences in modifying the result, stratified analyses were conducted based on age group, sex, race, poverty group, and education level.

To validate the robustness of our result, several sensitivity analyses were performed as follows: (1) Participants with cardiovascular disease (*n* = 1349) were excluded to avoid potential confounding effects; (2) the survey cycle was enrolled as another covariate to reassess the association of cardiovascular health metrics with depression and anxiety.

All analyses in the present study were performed by *R 4.2.3* software. The “survey” package was leveraged to process the weighted data. A two‐tailed *p* < 0.05 was considered statistically significant.

## 3 Results

### 3.1 Baseline Characteristics

Of the 13 028 participants enrolled in our study, 1206 participants (9.26%) exhibited depression symptoms and 3326 participants (25.53%) exhibited anxiety status. Table 1 demonstrated the baseline characteristics of the weighted population by the category of LE8. Compared to participants with high LE8 scores, those with low LE8 scores tended to be older, female, low educated, solitary, and poor. Additionally, participants with low LE8 were more likely to be companies with depression symptoms (weighted prevalence: 17.88%) and anxiety status (weighted prevalence: 35.87%) than moderate LE8 group (weighted prevalence: depression: 6.77%; anxiety: 24.48%) and high LE8 group (weighted prevalence: depression: 1.77%; anxiety: 20.03%). Table S1 described the distribution of LE8 components in the depression group and non‐depression group. For individual elements, depression participants exhibited inferior scores on diet, sleep health, nicotine exposure, physical activity, blood lipids, blood glucose, and BMI. Table S2 compared the distribution of LE8 components in the anxiety group and non‐anxiety group, in which anxiety participants demonstrated lower scores on diet, physical activity, nicotine exposure, sleep health, BMI, and blood lipids. These findings revealed that participants with depression or anxiety may have difficulty adhering to behaviors and factors that were conducive to cardiovascular health.

**Table 1:** Baseline characteristics of participants according to cardiovascular health (LE8) category.

Characteristics	Low LE8 (*n* = 2654)	Moderate LE8 (*n* = 8479)	High LE8 (*n* = 1895)	*p* value
Age (mean ± SE)^a^	52.00 ± 14.70	47.74 ± 16.87	40.51 ± 15.71	< 0.001
Sex (%)^b^				< 0.001
Male	15 234 522 (51.24)	57 999 439 (51.54)	13 145 796 (40.76)	
Female	14 495 918 (48.76)	54 529 157 (48.46)	19 107 195 (59.24)	
Race (%)				< 0.001
Mexican American	2 260 755 (7.60)	9 602 365 (8.53)	1 913 761 (5.93)	
Other Hispanic	1 417 180 (4.77)	6 138 568 (5.46)	1 393 747 (4.32)	
Non‐Hispanic White	20 557 507 (69.15)	78 734 013 (69.97)	24 450 235 (75.81)	
Non‐Hispanic Black	4 394 861 (14.78)	11 568 176 (10.28)	1 836 140 (5.69)	
Other race/multiracial	1 100 136 (3.70)	6 485 474 (5.76)	2 659 108 (8.24)	
Education level (%)				< 0.001
< High school	2 902 021 (9.76)	5 880 002 (5.23)	602 276 (1.87)	
High school	15 129 887 (50.91)	40 339 916 (35.89)	4 842 931 (15.02)	
> High school	11 688 404 (39.33)	66 185 393 (58.88)	26 807 783 (83.12)	
Marital (%)				0.049
Coupled	17 877 555 (60.15)	72 525 368 (64.47)	20 672 653 (64.11)	
Single or separated	11 843 717 (39.85)	39 961 455 (35.53)	11 573 292 (35.89)	
Poverty (mean ± SE)	2.46 ± 1.58	3.03 ± 1.63	3.60 ± 1.59	< 0.001
LE8 total score (mean ± SE)	41.86 ± 6.34	64.88 ± 8.34	86.67 ± 5.09	< 0.001
LE8 health behavior score	34.72 ± 15.51	60.90 ± 17.24	85.29 ± 9.88	< 0.001
LE8 health factor score	49.01 ± 15.18	68.86 ± 15.80	88.04 ± 10.10	< 0.001
Depression status (%)				< 0.001
Non‐depression	24 413 912 (82.12)	104 906 570 (93.23)	31 682 405 (98.23)	
Depression	5 316 528 (17.88)	7 622 026 (6.77)	570 586 (1.77)	
Anxiety status (%)				< 0.001
Non‐anxiety	19 066 613 (64.13)	84 982 532 (75.52)	25 793 246 (79.97)	
Anxiety	10 663 827 (35.87)	27 546 064 (24.48)	6 459 744 (20.03)	

Abbreviations: LE8, Life's Essential 8; SE, standard error.

^a^ Continuous variables were presented as weighted mean and standard errors.

^b^ Categorical variables were presented as weighted participant numbers and percentages.

### 3.2 Cardiovascular Health Metrics and Depression

**Figure 2:**
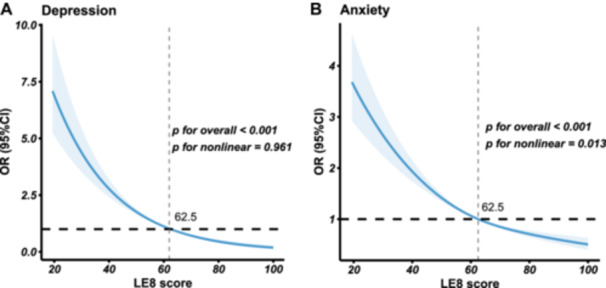
Restricted spline curves of LE8 score for depression (A) and anxiety (B). The restrict curves of weighted logistic regression models were adjusted for age, sex, race, education level, marital status, and poverty status. CI, confidence interval; LE8, Life's Essential 8; OR, adjusted odds ratio.

The prevalence of depression was found to be lower among participants with high LE8 total scores (1.77%) and moderate LE8 total scores (6.77%), in contrast to those with low LE8 total scores (17.88%). The weighted logistic regression model illustrated participants with moderate (OR, 95% CI: 0.36, 0.29–0.43) and high (OR, 95% CI: 0.08, 0.05–0.13) LE8 score were progressively less likely to develop depression compared to those with low LE8 score (*p* for trend < 0.001) (Table 2). In addition, better cardiovascular health with per 10‐unit increase of LE8 total score was associated with a 39% reduced risk of depression occurrence (OR, 95% CI: 0.61, 0.58–0.65) (Table 2). RCS showed that the LE8 score had a linear association with depression (*p* for nonlinear = 0.961) (Figure 2A). A similar trend was also observed in the association of depression with both the LE8 health behavior score (*p* for trend < 0.001) and LE8 health factor score (*p* for trend < 0.001) (Table 2). For per 10‐unit increase of LE8 health behavior score and LE8 health factor score, the odds of depression were 0.70 (95% CI: 0.67–0.73) and 0.87 (95% CI: 0.83–0.92), respectively (Table 2).

**Table 2:** Association between cardiovascular health (LE8) and occurrence of depression and anxiety.

Characteristics	Depression^a^			Anxiety^a^		
	OR (95% CI)	*p* value	*p* for trend	OR (95% CI)	*p* value	*p* for trend
LE8 total score			< 0.001			< 0.001
Low	Ref.			Ref.		
Moderate	0.36 (0.29–0.43)	< 0.001		0.57 (0.50–0.65)	< 0.001	
High	0.08 (0.05–0.13)	< 0.001		0.37 (0.32–0.44)	< 0.001	
LE8 health behavior score			< 0.001			< 0.001
Low	Ref.			Ref.		
Moderate	0.38 (0.31–0.46)	< 0.001		0.61 (0.53–0.71)	< 0.001	
High	0.15 (0.10–0.21)	< 0.001		0.47 (0.40–0.56)	< 0.001	
LE8 health factor score			< 0.001			< 0.001
Low	Ref.			Ref.		
Moderate	0.75 (0.58–0.97)	0.028		0.82 (0.73–0.91)	< 0.001	
High	0.52 (0.39–0.68)	< 0.001		0.71 (0.62–0.80)	< 0.001	
LE8 total score^b^	0.61 (0.58–0.65)	< 0.001		0.80 (0.77–0.82)	< 0.001	
LE8 health behavior score^b^	0.70 (0.67–0.73)	< 0.001		0.85 (0.82–0.87)	< 0.001	
LE8 health factor score^b^	0.87 (0.83–0.92)	< 0.001		0.93 (0.91–0.96)	< 0.001	

Abbreviations: CI, confidence interval; LE8, Life's Essential 8; OR, odds ratio; Ref., reference.

^a^ Adjusted for age, sex, race, marital status, poverty, and education.

^b^ Per 10‐unit increase.

The relationship between single LE8 components and depression was delineated in Supporting Information S1: Figure S1. The prevalence of depression was progressively lower in participants who scored higher on diet, sleep health, nicotine exposure, physical activity, blood lipids, blood glucose, and BMI (*p* for trend < 0.001). However, a similar trend was not found with the blood pressure (*p* for trend = 0.830).

### 3.3 Cardiovascular Health Metrics and Anxiety

Participants with high LE8 total scores (20.03%) and moderate LE8 total scores (24.48%) demonstrated a lower prevalence of anxiety compared to those with low LE8 total scores (35.87%). The weighted logistic regression model fully adjusted covariates illustrated that participants with moderate (OR, 95% CI: 0.57, 0.50–0.65) and high (OR, 95% CI: 0.37, 0.32–0.44) LE8 score were progressively less likely to develop anxiety compared to those with low LE8 score (*p* for trend < 0.001) (Table 2). For better cardiovascular health with a per 10‐unit increase of LE8 total score, the prevalence of anxiety could be decreased by 20% (OR, 95% CI: 0.80, 0.77–0.82) (Table 2). RCS demonstrated a nonlinear association between LE8 and anxiety (*p* for nonlinear = 0.013) (Figure 2B). Additionally, LE8 health behavior score and health factor score were also negatively associated with the occurrence of anxiety. Per 10‐unit increase of LE8 health behavior score and LE8 health factor score were associated with 15% (95% CI: 0.82–0.87) and 7% (95% CI: 0.91–0.96) reduced risk of anxiety, respectively (Table 2).

The relationship between single LE8 components and anxiety was delineated in Supporting Information S1: Figure S2. The prevalence of anxiety was progressively lower in participants who scored higher on diet, sleep health, nicotine exposure, physical activity, blood lipids, blood glucose, and BMI.

### 3.4 Subtype and Sensitivity Analysis

**Figure 3:**
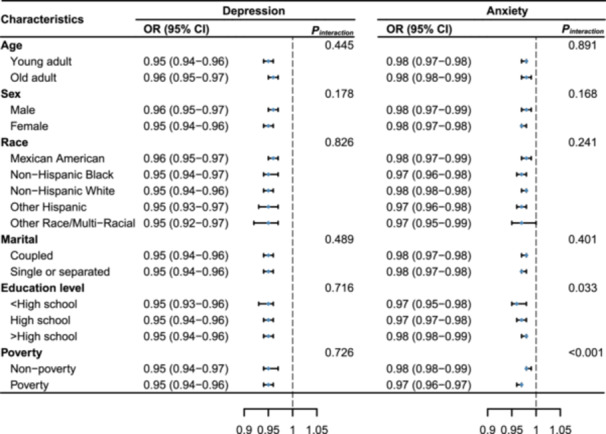
Stratification analysis of the correlations of CVH using the LE8 with depression and anxiety. Models were adjusted for age, sex, race, education level, marital status, and poverty status. CI, confidence interval; CVH, cardiovascular health; LE8, Life's Essential 8; OR, odds ratio; *p*_interaction_, *p* for interaction.

The subgroup analysis was demonstrated in Figure 3. The negative association between LE8 and depression was found in all subgroups and no interactions were observed. For subtype analysis between LE8 and anxiety, interactions between LE8 and education level (*p* for interaction = 0.033) and poverty (*p* for interaction < 0.001) were observed. The negative relationship between LE8 and anxiety was stronger in participants with low education levels and poverty status.

Several sensitivity analyses were also conducted in our study. The results were similar when we repeated the main analysis after enrolling the survey cycle as a covariate (Supporting Information S1: Figure S3 and S4). Furthermore, to avoid the bias of CVD, we repeated the main analysis after removing all CVD participants and the result remained significant and similar to the previous result (Figure S5 and S6).

## 4 Discussion

In the nationally representative cross‐sectional research, better cardiovascular health defined by LE8 total score was found associated with both lower occurrence of depression and anxiety. The RCS analysis showed a nonlinear association between LE8 and anxiety. Furthermore, the negative association remained significant in subgroup analysis and sensitivity analysis.

To our knowledge, this was the first research to examine the association of cardiovascular health defined by LE8 with depression and anxiety. Previous evidence has illustrated that behavioral and biological processes contributing to CVD were linked to depression and anxiety, which served as the foundation of our study [12, 22, 23, 24]. Other recent studies, including the English Longitudinal Study of Ageing (ELSA, *n* = 3231), the GAZEL cohort (*n* = 20 625), and NHANES (*n* = 14 561), evaluated the association of baseline cardiovascular health metrics and the presence of depression symptoms [12, 25, 26]. A cohort study involving 9962 Chinese participants found a negative correlation between anxiety and the number of ideal health metrics [27]. In line with our findings, participants in these cohorts with favorable cardiovascular health exhibited a lower prevalence of depression and anxiety. However, the cardiovascular health metrics used in these studies were based on LS7, which could only be displayed as a categorical variable. Limitations of the scoring algorithm and inadequacies of the metrics rendered LS7 less sensitive in assessing cardiovascular health [13]. Pleasingly, the updated and modified LE8 allowed the assessment of the full scope of metrics and has been regarded as the superior representation of cardiovascular health [13]. This study contributed remarkable evidence to the relationship between cardiovascular health and mental disorders by utilizing LE8 to define cardiovascular health.

The underlying mechanisms connecting cardiovascular health and mental disorders tended to be intricate and multifactorial. Impaired cardiovascular health was frequently accompanied by elevated levels of inflammatory factors, which was also one feature of depression and anxiety [28, 29, 30, 31]. Other possible mechanisms that may explain the association between cardiovascular health and depression included hypothalamic‐pituitary‐adrenal axis (HPA) dysfunction and neurohormonal imbalance, which have been identified in both depressed patients and patients with cardiovascular disease [29, 32]. However, further investigation is needed to fully understand the exact mechanisms underlying the link between cardiovascular health and mental disorders.

In the subgroup analysis, the relationship between LE8 and anxiety was more pronounced among individuals with lower education levels and those living in poverty. We noted that these populations often encounter significant barriers to accessing health resources, experience heightened psychological stress, and generally have lower baseline levels of healthy behaviors. Consequently, even modest improvements in health practices, as encouraged by LE8, could lead to substantial benefits in mental health.

Intriguingly, the negative association between cardiovascular health metrics and mental disorders appeared to be more significant for the LE8 health behavior score than the LE8 health factor score. This inconsistency could be attributed to the distinct time‐frame nature in which the two sets of variables were acquired. The majority of LE8 health behaviors in NHANES data only reflected the recent status, with diet, physical activity, and sleep health mirroring the status of the past 24 h, 7 days, and 1 month, respectively. However, the LE8 health factors (blood pressure, blood glucose, blood lipids, and BMI) needed more time to develop and change. Therefore, the relationship between LE8 health behavior score and mental disorders was more immediate and likely to be captured in cross‐sectional cohorts such as NHANES [12].

Prior evidence has examined the association of depression with individual LE8 health behavior indicators, including diet, physical activity, nicotine exposure, and sleep health. Diet was proven to be closely related to depression, but most studies focused only on specific dietary patterns or nutrients, such as the Mediterranean diet, minerals, vitamins, and so on [33, 34, 35]. To explore the association between diet and depression from a more macroscopic perspective, Wang et al. leveraged the HEI‐2015 score to reflect diet quality [36]. Consistent with our results, optimal HEI status was associated with lower odds of depression and anxiety, indicating that maintaining a healthy diet could reduce the risk of depression [36, 37]. Numerous prospective cohort studies suggested that physical activity could decrease the risk of depression and anxiety, which deciphered a potentially modifiable target for these mental disorders' prevention [38, 39]. Nicotine exposure often appeared as a comorbidity of mental disorders, with smoking being more prevalent and more difficult to quit in patients with depression or anxiety [40, 41]. Previous studies have reported an increased presence of depression and anxiety status in smokers compared to nonsmokers, which was concordant with our current result [42]. The effect of nicotine on neurotransmitter activity, which was suggested to be responsible for depressive symptoms in the monoamine hypothesis, provided a plausible mechanism for the relationship between improved nicotine exposure score and decreased risk of depression [42]. The bidirectional relationship between sleep health and mental disorders has been extensively explored, and their negative association was also manifested in the abovementioned results [43]. Given the longitudinal association between insomnia and the risk of depression and anxiety, sleep health has become a critical target for their prevention and treatment [43].

For individual LE8 health factor indicators, except blood pressure, all other metrics were each observed as negatively associated with depression. The relationship between blood pressure and depression has been controversial for a long time. A study found that an increased CES‐D score (used to assess depression symptoms) was associated with lower systolic blood pressure in the overall population, but only with lower rates of hypertension in men [44]. Globally, cross‐sectional studies showed a negative association between blood pressure and depression, whereas longitudinal studies demonstrated a positive association [45, 46]. In our study, the relationship between blood pressure and depression was not clear. Further exploration is needed to fully understand the relationship between blood pressure and depression, as well as its underlying mechanisms.

Although a nationally representative cohort was utilized to explore the association between LE8 and the prevalence of depression and anxiety, several limitations still existed in our research. First, several LE8 metrics were collected from self‐reported questionnaires, which can be prone to selection bias and desirability bias. Second, depression and anxiety were obtained based on simple self‐reported scales, which were not fully accurate in diagnosing diseases. Although the PHQ‐9 and similar scales are effective for identifying individuals at risk of depression and anxiety, they do not provide a definitive diagnosis. These scales are designed to screen for symptoms that may warrant further clinical evaluation but could not be interpreted as equivalent to a clinical diagnosis made by a healthcare professional. Third, while we controlled for known confounders in our research, potentially unknown covariates may have had an impact on the results. Finally, the cross‐sectional study design impeded us from concluding the temporality and causality of the association between LE8 and the risk of depression and anxiety.

## 5 Conclusions

In the nationally representative cross‐sectional research, cardiovascular health, as defined by the LE8 score, was found to have an independent and negative association with the prevalence of depression and anxiety. These findings remained consistent across all subgroups and sensitivity analyses. In summary, our research indicated that cardiovascular health was closely associated with mental health and the prospective beneficial role of LE8 for depression and anxiety prevention deserves further exploration.

## Author Contributions

Yudi Xu contributed to the study design, data analysis, visualization, and paper writing. Wenjing Ning and Yuyuan Zhang contributed to the study design and paper revisiting. Yanjie Jia and Xinwei Han contributed to project oversight and paper revisiting. Yuhao Ba, Huimin Liu, Long Liu, Libo Wang, Chunguang Guo, Hui Xu, Siyuan Weng, Zhaokai Zhou, Zongao Cai, Hongxuan Ma, and Ge Zhang contributed to paper revisiting. All authors approved this manuscript.

## Ethics Statement

The authors have nothing to report.

## Consent

The authors have nothing to report.

## Conflicts of Interest

The authors declare no conflicts of interest.

## Supporting information

Supporting information.

Supporting information.

Supporting information.

## Data Availability

The National Health and Nutrition Examination Survey data set is publicly available at the National Center for Health Statistics of the Center for Disease Control and Prevention (https://www.cdc.gov/nchs/nhanes/index.htm).
