# Supplementary material for: Associations Between Cardiovascular Health (Life's Essential 8) and Mental Disorders
Source: Clin Cardiol. 2024 Sep 24;47(9):e70019. doi: 10.1002/clc.70019 (PMC11420513; doi:10.1002/clc.70019)
Supplement: Supplementary file 1 — Supporting information. [file CLC-47-e70019-s002.docx]

**Supplementary Figure**

- **Figure S1**
- **Figure S2**
- **Figure S3**
- **Figure S4**
- **Figure S5**
- **Figure S6**


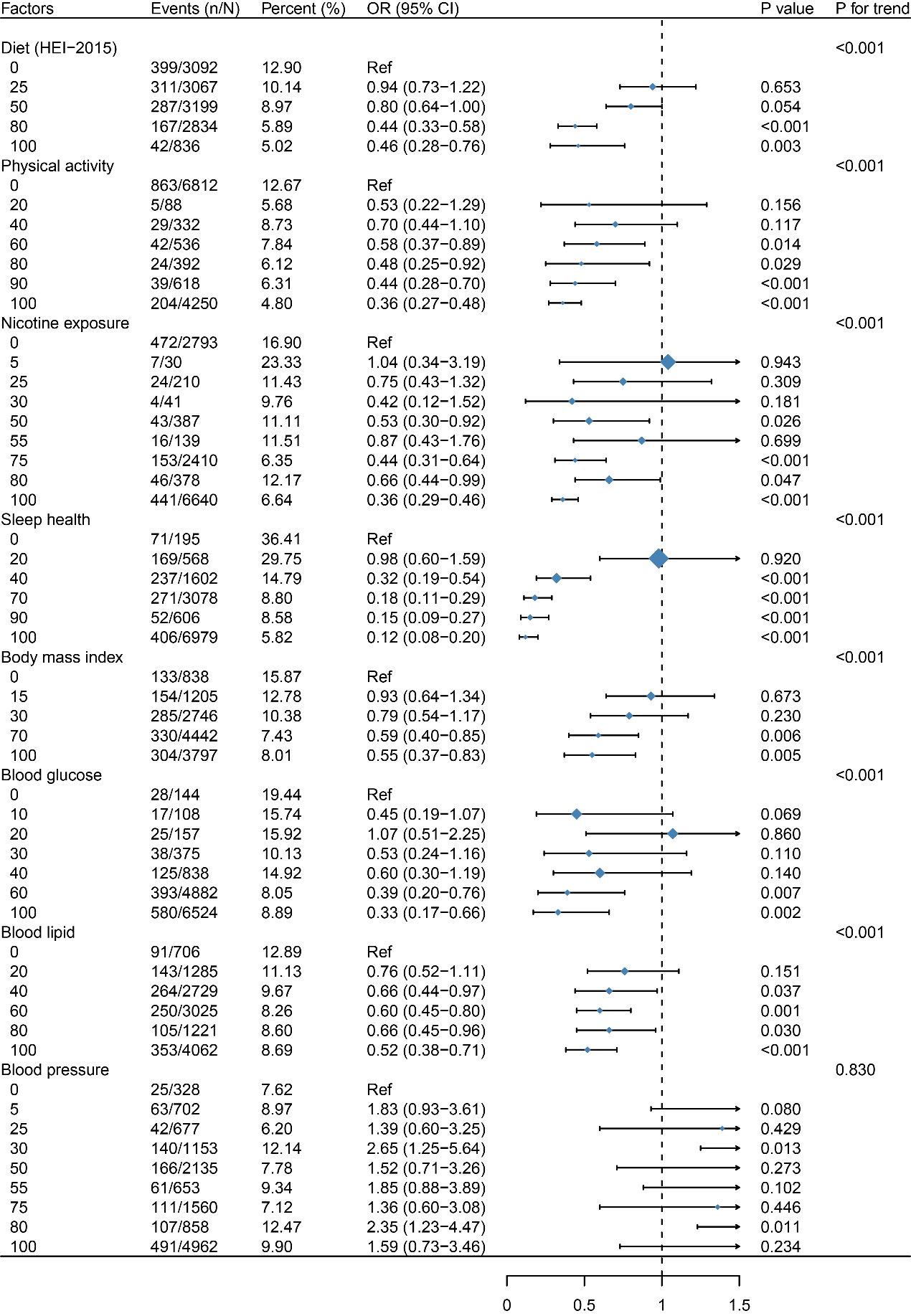


**Figure S1: Association between individual LE8 components and risk of depression.**

OR, odds ratio; CI, confidence interval; LE8, life’s essential 8; Ref, reference.

The model adjusted for age, sex, race, marital status, poverty, and education.


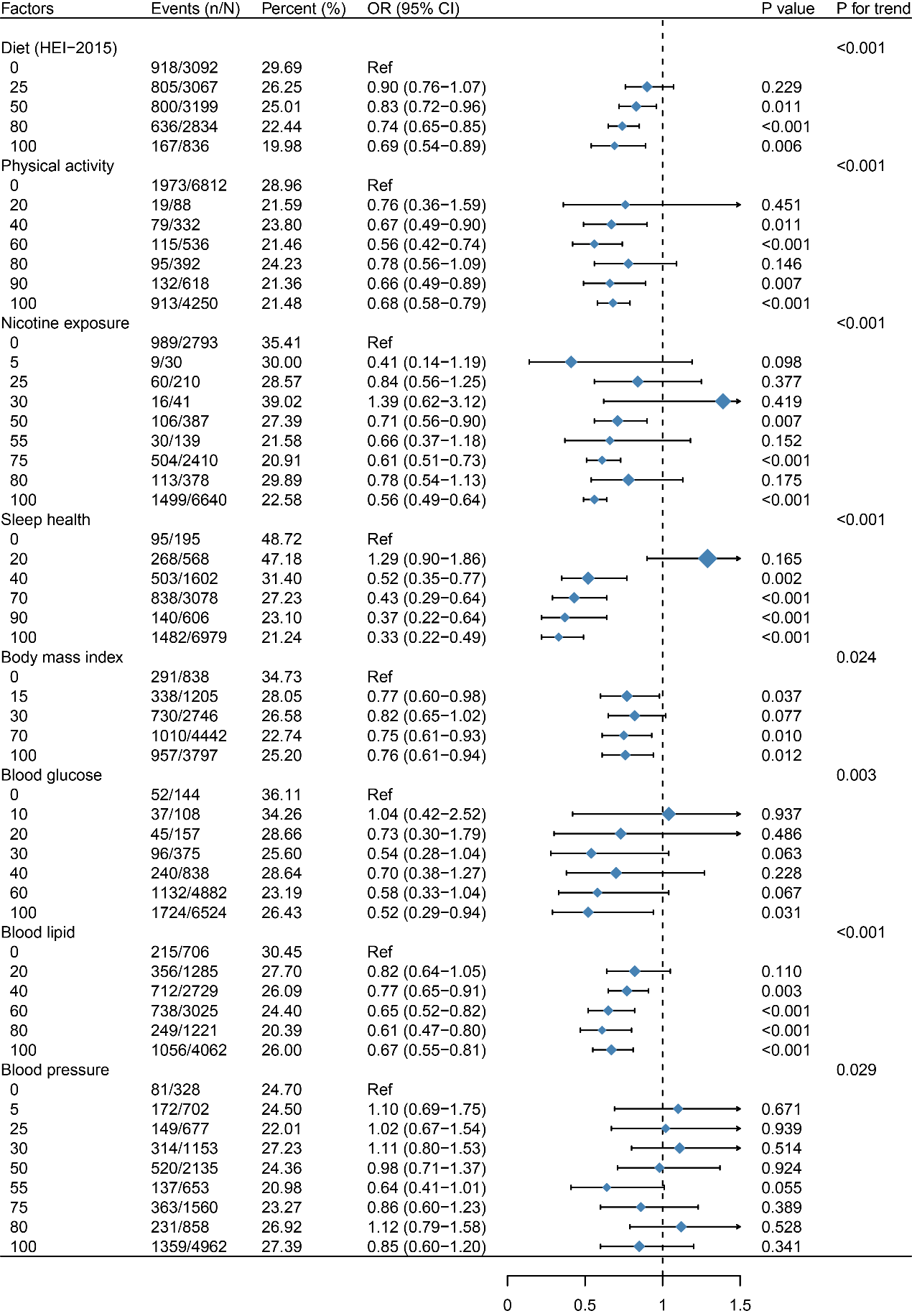


**Figure S2: Association between individual LE8 components and risk of anxiety.**

OR, odds ratio; CI, confidence interval; LE8, life’s essential 8; Ref, reference.

The model adjusted for age, sex, race, marital status, poverty, and education.


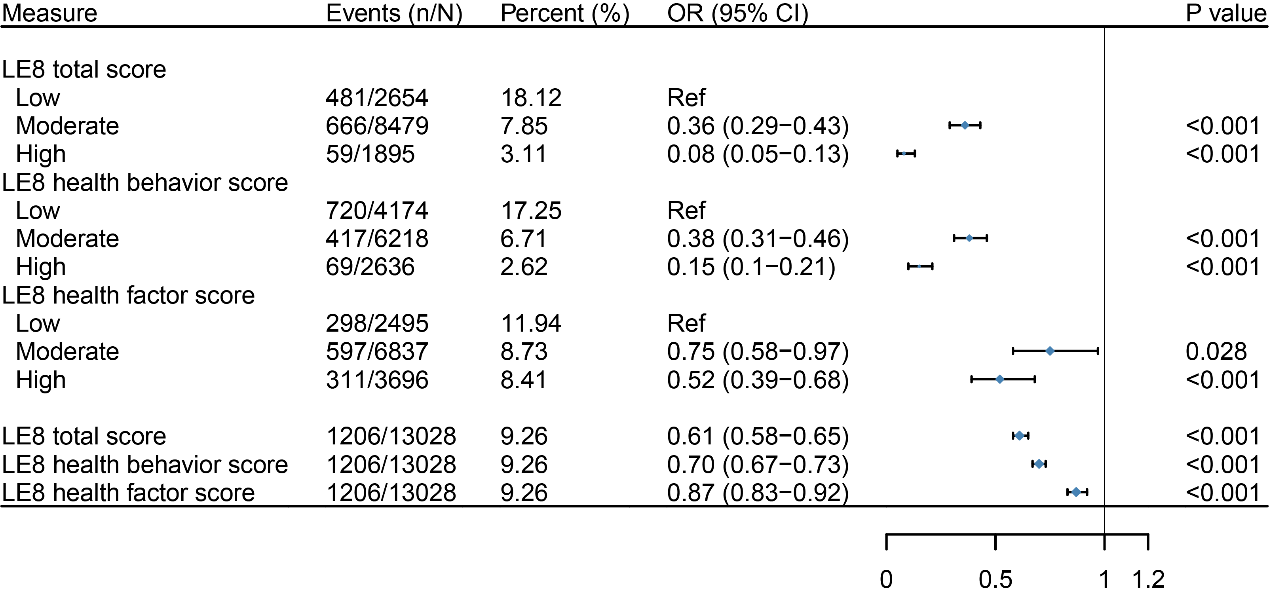


**Figure S3: The association between cardiovascular health (CVH) metrics with depression after the survey cycle was enrolled as another covariate.**

a: Adjusted for age, sex, race, marital status, poverty, education, and survey cycle.

The continuous variables were represented by per 10-unit increase.


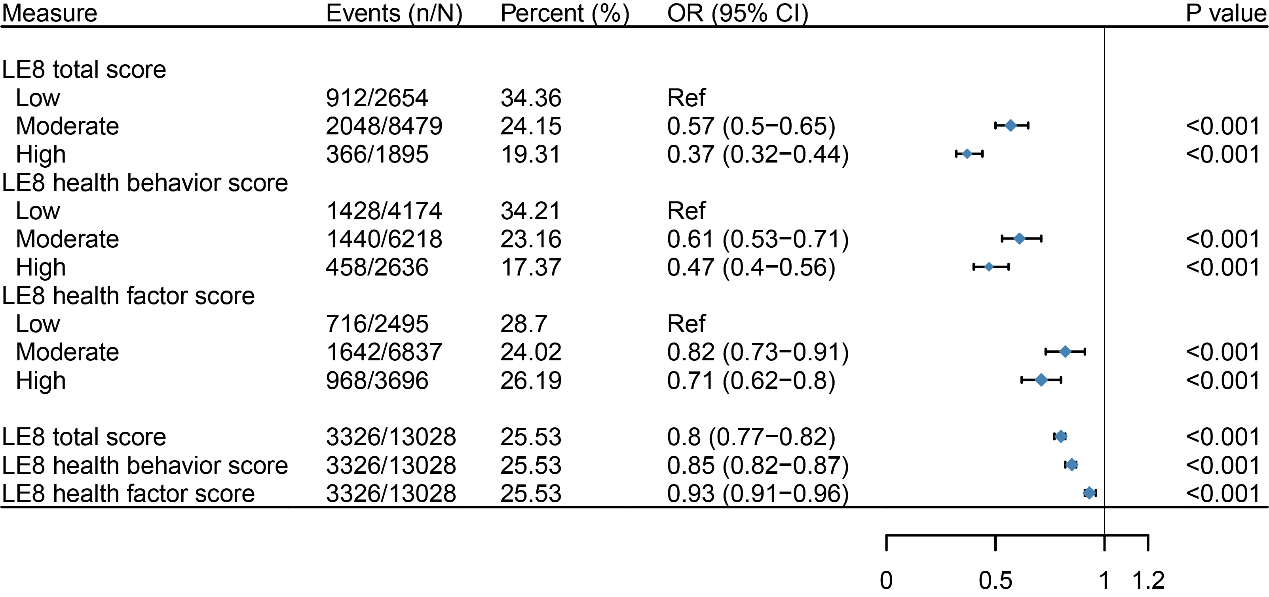


**Figure S4: The association between cardiovascular health (CVH) metrics with anxiety after the survey cycle was enrolled as another covariate.**

a: Adjusted for age, sex, race, marital status, poverty, education, and survey cycle.

The continuous variables were represented by per 10-unit increase.


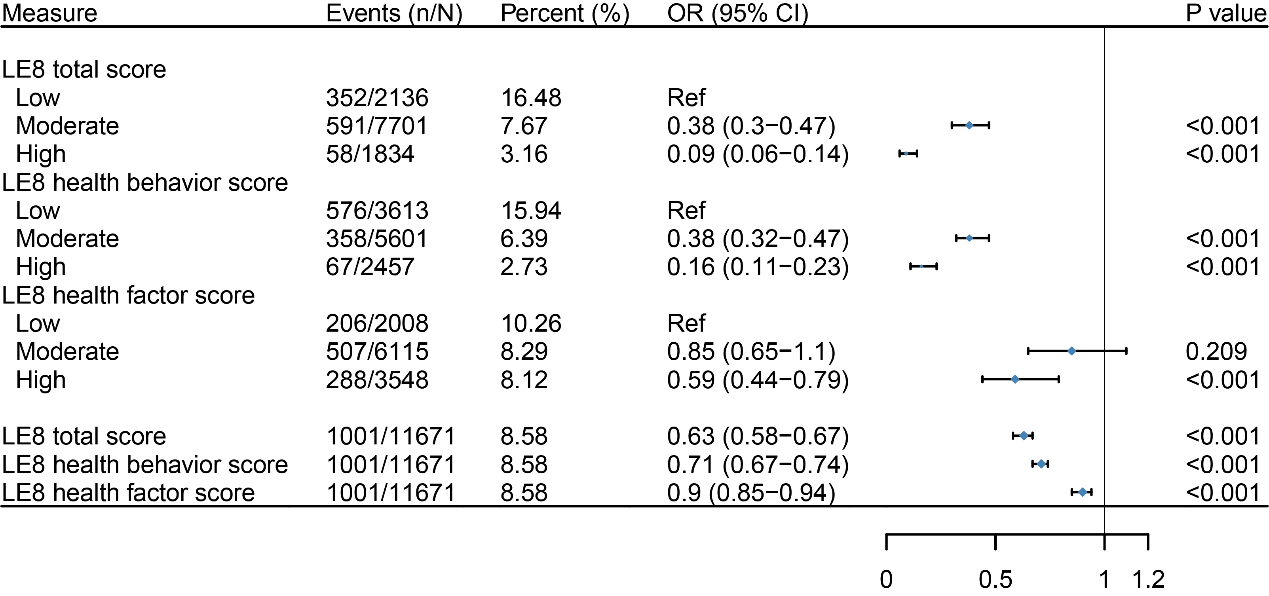


**Figure S5: The association between cardiovascular health (CVH) metrics with depression after deletion of cardiovascular diseases (CVD) cases (n = 1,349).**

a: Adjusted for age, sex, race, marital status, poverty, and education.

The continuous variables were represented by per 10-unit increase.


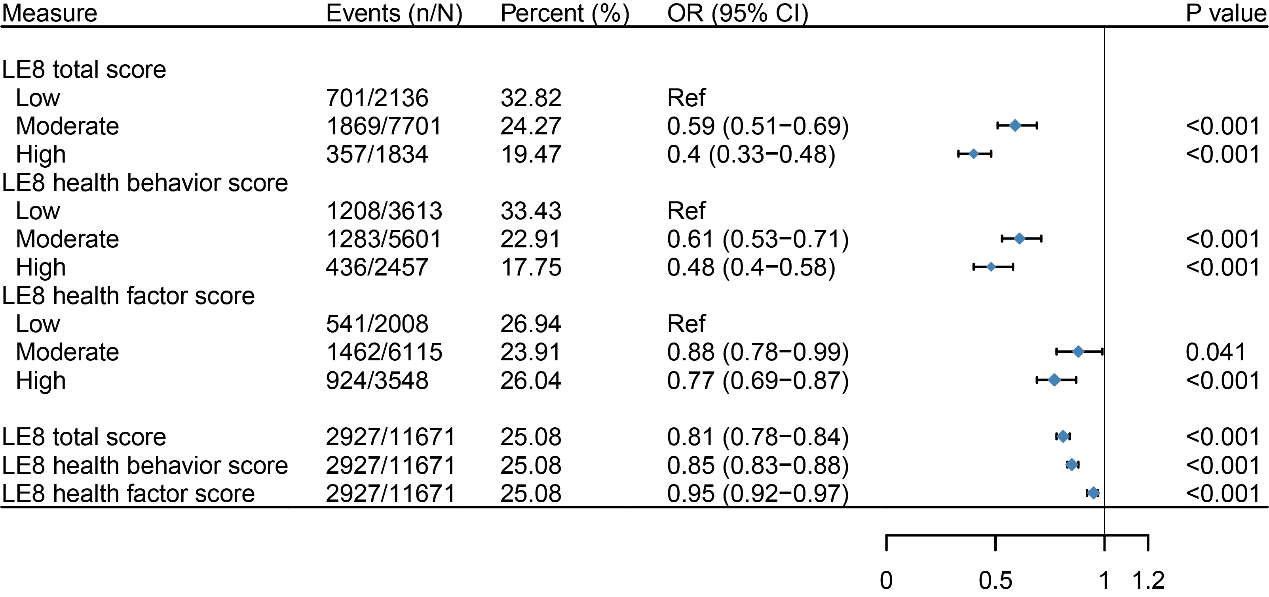


**Figure S6: The association between cardiovascular health (CVH) metrics with anxiety after deletion of cardiovascular diseases (CVD) cases (n = 1,349).**

a: Adjusted for age, sex, race, marital status, poverty, and education.

The continuous variables were represented by per 10-unit increase.
