# Supplementary material for: Associations Between Cardiovascular Health (Life's Essential 8) and Mental Disorders
Source: Clin Cardiol. 2024 Sep 24;47(9):e70019. doi: 10.1002/clc.70019 (PMC11420513; doi:10.1002/clc.70019)
Supplement: Supplementary file 2 — Supporting information. [file CLC-47-e70019-s001.docx]

**Assessment of cardiovascular health metrics**

According to the American Heart Association (AHA), the detailed information of LE8 were listed as follows[1]:

The calculation of the LE8 score involves eight key metrics, each of which is scored according to specific criteria, with the final score being the average of all eight components. First, for diet, the HEI-2015 score is used, with adherence to a healthy eating pattern determining the score, up to a maximum of 100 points. Second, physical activity (PA) is scored based on the number of minutes of moderate to vigorous activity per week, with 150 minutes or more earning a full 100 points. Nicotine exposure considers smoking, e-cigarette use, and secondhand smoke exposure; those who have never smoked receive 100 points, while current smokers receive 0 points. Sleep health is scored based on average hours of sleep per night, with 7 to 9 hours earning a full 100 points, while insufficient or excessive sleep results in deductions. Body Mass Index (BMI) is calculated based on weight and height, with a BMI under 25 earning 100 points, and a BMI of 40 or higher earning 0 points. Blood lipids are scored using non-high-density lipoprotein cholesterol (non-HDL-C) levels, with levels under 130 mg/dL receiving 100 points and levels of 220 mg/dL or higher receiving 0 points. Blood glucose is scored based on fasting blood glucose (FBG) or hemoglobin A1c (HbA1c) levels, with FBG under 100 mg/dL or HbA1c under 5.7% earning 100 points, while diabetes patients with HbA1c levels of 10.0% or higher receive 0 points. Finally, blood pressure (BP) is scored based on systolic and diastolic levels, with levels under 120/80 mm Hg receiving 100 points and levels of 160/100 mm Hg or higher receiving 0 points. If blood pressure or lipid levels are controlled by medication, 20 points are deducted from the score. The scores for all eight metrics are then averaged to calculate the final LE8 score, which ranges from 0 to 100 points, with higher scores indicating better cardiovascular health.

1 Lloyd-Jones, D.M., Allen, N.B., Anderson, C.A.M., Black, T., Brewer, L.C., Foraker, R.E., Grandner, M.A., Lavretsky, H., Perak, A.M., Sharma, G. *et al.* (2022) Life's Essential 8: Updating and Enhancing the American Heart Association's Construct of Cardiovascular Health: A Presidential Advisory From the American Heart Association. *Circulation*, **146**, e18-e43.
