# Supplementary material for: Associations Between Cardiovascular Health (Life's Essential 8) and Mental Disorders
Source: Clin Cardiol. 2024 Sep 24;47(9):e70019. doi: 10.1002/clc.70019 (PMC11420513; doi:10.1002/clc.70019)
Supplement: Supplementary file 3 — Supporting information. [file CLC-47-e70019-s003.docx]

Supplementary Tables

- Table S1
- Table S2

**Table S1: Baseline characteristics of single LE8 components in participants with and without depression.**

| **Factors** | **Points** | **Non-depression Participants** | **Depression Participants** | ***P* value** |
| --- | --- | --- | --- | --- |
| **Diet (HEI-2015) (%)** |  |  |  | <0.001 |
| 1-24 | 0 | 37,436,507 (23.25) | 4,389,467 (32.49) |  |
| 25-49 | 25 | 36,842,406 (22.88) | 3,763,404 ( 27.86) |  |
| 50-74 | 50 | 39,136,601 ( 24.31) | 3,311,976 (24.52) |  |
| 75-94 | 80 | 36,418,443 (22.62) | 1,558,800 (11.54) |  |
| >=95 | 100 | 11,168,929 (6.94) | 485,492 (3.59) |  |
| **Physical activity (min/week)** |  |  |  | <0.001 |
| 0 | 0 | 69,967,531 (43.46) | 9,320,464 (68.99) |  |
| 1-29 | 20 | 1,058,734 (0.66) | 62,047 (0.46) |  |
| 30-59 | 40 | 4,566,266 (2.84) | 335,078 (2.48) |  |
| 60-89 | 60 | 7,468,855 (4.64) | 484,673 (3.59) |  |
| 90-119 | 80 | 5,386,361 (3.35) | 300,847 (2.23) |  |
| 120-149 | 90 | 8,907,486 (5.53) | 442,439 (3.28) |  |
| >=150 | 100 | 63,647,654 (39.53) | 2,563,591 (18.98) |  |
| **Nicotine exposure** |  |  |  | <0.001 |
| Current smoker | 0 | 30,684,113 (19.06) | 5,498,818 (40.70) |  |
| Former smoker and second-hand exposure (quit <1 year) | 5 | 309,722 (0.19) | 58,842 (0.44) |  |
| Former smoker (quit <1 year) | 25 | 2,966,850 (1.84) | 324,731 (2.40) |  |
| Former smoker and second-hand exposure (quit 1–<5 years) | 30 | 558,971 (0.35) | 33,311 (0.25) |  |
| Former smoker (quit 1–<5 year) | 50 | 5,673,630 (3.52) | 466,554 (3.45) |  |
| Former smoker and second-hand exposure (quit ≥5 years) | 55 | 1,476,489 (0.92) | 176,543 (1.31) |  |
| Former smoker (quit ≥5years) | 75 | 29,962,539 (18.61) | 1,682,646 (12.46) |  |
| Never smoker, but second-hand exposure | 80 | 4,123,684 (2.56) | 524,085 (3.88) |  |
| Never smoker | 100 | 85,246,889 (52.95) | 4,743,609 (35.11) |  |
| **Sleep health (average hours/night)** |  |  |  | <0.001 |
| <4 | 0 | 1,078,611 (0.67) | 652,516 (4.83) |  |
| 4–<5 | 20 | 4,587,969 (2.85) | 2,121,787 (15.71) |  |
| 5–<6 or ≥10 | 40 | 15,727,323 (9.77) | 2,562,344 (18.97) |  |
| 6–<7 | 70 | 37,792,915 (23.47) | 2,833,069 (20.97) |  |
| 9–<10 | 90 | 7,425,781 (4.61) | 543,907 (4.03) |  |
| 7–<9 | 100 | 94,390,288 (58.63) | 4,795,517 (35.50) |  |
| **Body mass index (kg/m2)** |  |  |  | <0.001 |
| ≥40.0 | 0 | 8,935,274 (5.55) | 1,357,129 (10.05) |  |
| 35.0–39.9 | 15 | 13,299,721 (8.26) | 1,671,611 (12.37) |  |
| 30.0–34.9 | 30 | 32,215,622 (20.01) | 2,986,211 (22.11) |  |
| 25.0–29.9 | 70 | 56,305,912 (34.97) | 3,881,645 (28.73) |  |
| <25 | 100 | 50,246,358 (31.21) | 3,612,543 (26.74) |  |
| **Blood lipids (mg/dL)** |  |  |  | 0.008 |
| Non-HDL ≥220 (or 190–219 treated) | 0 | 7,710,378 (4.79) | 1,015,801 (7.52) |  |
| Non-HDL 190–219 (or 160–189 treated) | 20 | 15,705,283 (9.75) | 1,593,314 (11.79) |  |
| Non-HDL 160–189 (or 130–159 treated) | 40 | 35,095,665 (21.80) | 2,985,356 (22.10) |  |
| Non-HDL 130–159 | 60 | 38,191,344 (23.72) | 2,999,410 (22.20) |  |
| Non-HDL <130 treated | 80 | 13,502,314 (8.39) | 940,839 (6.96) |  |
| Non-HDL <130 | 100 | 50,797,903 (31.55) | 3,974,419 (29.42) |  |
| **Blood glucose** |  |  |  | <0.001 |
| Diabetes with HbA1c ≥10.0 | 0 | 960,643 (0.60) | 241,572 (1.79) |  |
| Diabetes with Hb A1c 9.0–9.9 | 10 | 855,564 (0.53) | 99,655 (0.74) |  |
| Diabetes with HbA1c 8.0–8.9 | 20 | 1,343,925 (0.83) | 302,836 (2.24) |  |
| Diabetes with HbA1c 7.0–7.9 | 30 | 3,197,710 (1.99) | 344,602 (2.55) |  |
| Diabetes with HbA1c <7.0 | 40 | 6,970,478 (4.33) | 1,023,198 (7.57) |  |
| No diabetes and FBG 100–125 (or HbA1c 5.7–6.4)(prediabetes) | 60 | 56,118,816 (34.86) | 4,541,269 (33.62) |  |
| No history of diabetes and FBG <100 (or HbA1c <5.7) | 100 | 91,555,751 (56.87) | 6,956,007 (51.49) |  |
| **Blood pressure (SBP or DBP), mmHg** |  |  |  | 0.004 |
| ≥160 or ≥100 | 0 | 2,655,152 (1.65) | 188,510 (1.40) |  |
| 140–159 or 90–99 treated | 5 | 6,854,774 (4.26) | 595,581 (4.41) |  |
| 140–159 or 90–99 | 25 | 7,300,856 (4.53) | 502,981 (3.72) |  |
| 130–139 or 80–89 treated | 30 | 11,195,320 (6.95) | 1,390,227 (10.29) |  |
| 130–139 or 80–89 | 50 | 27,812,760 (17.27) | 2,006,940 (14.86) |  |
| 120–129/<80 treated | 55 | 7,193,709 (4.47) | 584,260 (4.32) |  |
| 120–129/<80 | 75 | 21,340,393 (13.25) | 1,344,463 (9.95) |  |
| <120/<80 treated | 80 | 9,727,813 (6.04) | 1,085,083 (8.03) |  |
| <120/<80 | 100 | 66,922,108 (41.57) | 5,811,095 (43.02) |  |

**Table S2: Baseline characteristics of single LE8 components in participants with and without anxiety.**

| **Factors** | **Points** | **Non-anxiety Participants** | **Anxiety Participants** | **P value** |
| --- | --- | --- | --- | --- |
| **Diet (HEI-2015) (%)** |  |  |  | <0.001 |
| 1-24 | 0 | 29,310,226 (22.57) | 12,515,748 (28.02) |  |
| 25-49 | 25 | 29,786,735 (22.94) | 10,819,076 (24.22) |  |
| 50-74 | 50 | 31,821,414 (24.51) | 10,627,163 (23.79) |  |
| 75-94 | 80 | 29,642,669 (22.83) | 8,334,575 (18.66) |  |
| >=95 | 100 | 9,281,349 (7.15) | 2,373,073 (5.31) |  |
| **Physical activity (min/week)** |  |  |  | <0.001 |
| 0 | 0 | 55,782,475 (42.96) | 23,505,519 ( 52.62) |  |
| 1-29 | 20 | 860,752 (0.66) | 260,029 (0.58) |  |
| 30-59 | 40 | 3,824,401 (2.95) | 1,076,943 (2.41) |  |
| 60-89 | 60 | 6,422,414 (4.95) | 1,531,114 (3.43) |  |
| 90-119 | 80 | 4,210,919 (3.24) | 1,476,289 (3.30) |  |
| 120-149 | 90 | 7,276,781 (5.60) | 2,073,144 (4.64) |  |
| >=150 | 100 | 51,464,650 (39.64) | 14,746,596 (33.01) |  |
| **Nicotine exposure** |  |  |  | <0.001 |
| Current smoker | 0 | 23,238,632 (17.90) | 12,944,298 ( 28.98) |  |
| Former smoker and second-hand exposure (quit <1 year) | 5 | 279,527 (0.22) | 89,038 (0.20) |  |
| Former smoker (quit <1 year) | 25 | 2,321,024 (1.79) | 970,557 (2.17) |  |
| Former smoker and second-hand exposure (quit 1–<5 years) | 30 | 340,145 (0.26) | 252,137 (0.56) |  |
| Former smoker (quit 1–<5 year) | 50 | 4,462,267 (3.44) | 1,677,917 (3.76) |  |
| Former smoker and second-hand exposure (quit ≥5 years) | 55 | 1,286,420 (0.99) | 366,612 (0.82) |  |
| Former smoker (quit ≥5years) | 75 | 24,899,952 (19.18) | 6,745,234 (15.10) |  |
| Never smoker, but second-hand exposure | 80 | 3,213,285 (2.47) | 1,434,484 (3.21) |  |
| Never smoker | 100 | 69,801,139 (53.76) | 20,189,359 (45.20) |  |
| **Sleep health (average hours/night)** |  |  |  | <0.001 |
| <4 | 0 | 890,378 (0.69) | 840,750 (1.88) |  |
| 4–<5 | 20 | 3,164,173 (2.44) | 3,545,582 (7.94) |  |
| 5–<6 or ≥10 | 40 | 12,546,802 (9.66) | 5,742,864 (12.86) |  |
| 6–<7 | 70 | 29,826,469 (22.97) | 10,799,514 (24.18) |  |
| 9–<10 | 90 | 5,956,163 (4.59) | 2,013,526 (4.51) |  |
| 7–<9 | 100 | 77,458,406 (59.66) | 21,727,399 (48.64) |  |
| **Body mass index (kg/m2)** |  |  |  | <0.001 |
| ≥40.0 | 0 | 6,891,297 (5.31) | 3,401,105 (7.61) |  |
| 35.0–39.9 | 15 | 11,001,725 (8.47) | 3,969,608 (8.89) |  |
| 30.0–34.9 | 30 | 26,313,460 (20.27) | 8,888,374 (19.90) |  |
| 25.0–29.9 | 70 | 45,804,873 (35.28) | 14,382,684 (32.20) |  |
| <25 | 100 | 39,831,037 (30.68) | 14,027,863 (31.40) |  |
| **Blood lipids (mg/dL)** |  |  |  | <0.001 |
| Non-HDL ≥220 (or 190–219 treated) | 0 | 6,030,580 (4.64) | 2,695,599 (6.03) |  |
| Non-HDL 190–219 (or 160–189 treated) | 20 | 12,574,696 (9.68) | 4,723,901 (10.58) |  |
| Non-HDL 160–189 (or 130–159 treated) | 40 | 28,058,880 (21.61) | 10,022,141 (22.44) |  |
| Non-HDL 130–159 | 60 | 31,178,823 (24.01) | 10,011,931 (22.41) |  |
| Non-HDL <130 treated | 80 | 11,682,459 (9.00) | 2,760,694 (6.18) |  |
| Non-HDL <130 | 100 | 40,316,954 (31.05) | 14,455,368 (32.36) |  |
| **Blood glucose** |  |  |  | 0.297 |
| Diabetes with HbA1c ≥10.0 | 0 | 778,154 (0.60) | 424,061 (0.95) |  |
| Diabetes with Hb A1c 9.0–9.9 | 10 | 640,081 (0.49) | 315,138 (0.71) |  |
| Diabetes with HbA1c 8.0–8.9 | 20 | 1,186,070 (0.91) | 460,692 (1.03) |  |
| Diabetes with HbA1c 7.0–7.9 | 30 | 2,727,419 (2.10) | 814,892 (1.82) |  |
| Diabetes with HbA1c <7.0 | 40 | 5,778,026 (4.45) | 2,215,650 (4.96) |  |
| No diabetes and FBG 100–125 (or HbA1c 5.7–6.4)(prediabetes) | 60 | 45,641,616 (35.15) | 15,018,469 (33.62) |  |
| No history of diabetes and FBG <100 (or HbA1c <5.7) | 100 | 73,091,025 (56.29) | 25,420,733 (56.91) |  |
| **Blood pressure (SBP or DBP), mmHg** |  |  |  | 0.032 |
| ≥160 or ≥100 | 0 | 2,085,873 (1.61) | 757,789 (1.70) |  |
| 140–159 or 90–99 treated | 5 | 5,583,793 (4.30) | 1,866,562 (4.18) |  |
| 140–159 or 90–99 | 25 | 5,865,557 (4.52) | 1,938,280 (4.34) |  |
| 130–139 or 80–89 treated | 30 | 9,287,805 (7.15) | 3,297,742 (7.38) |  |
| 130–139 or 80–89 | 50 | 22,153,254 (17.06) | 7,666,446 (17.16) |  |
| 120–129/<80 treated | 55 | 6,421,423 (4.95) | 1,356,546 (3.04) |  |
| 120–129/<80 | 75 | 17,257,567 (13.29) | 5,427,289 (12.15) |  |
| <120/<80 treated | 80 | 7,881,044 (6.07) | 2,931,851 (6.56) |  |
| <120/<80 | 100 | 53,306,074 (41.05) | 19,427,129 (43.49) |  |
